# Supplementary figures and images for: Causal effect of porphyria biomarkers on alcohol-related hepatocellular carcinoma through Mendelian Randomization
Source: PLoS One. 2024 Mar 20;19(3):e0299536. doi: 10.1371/journal.pone.0299536 (PMC10954128; doi:10.1371/journal.pone.0299536)

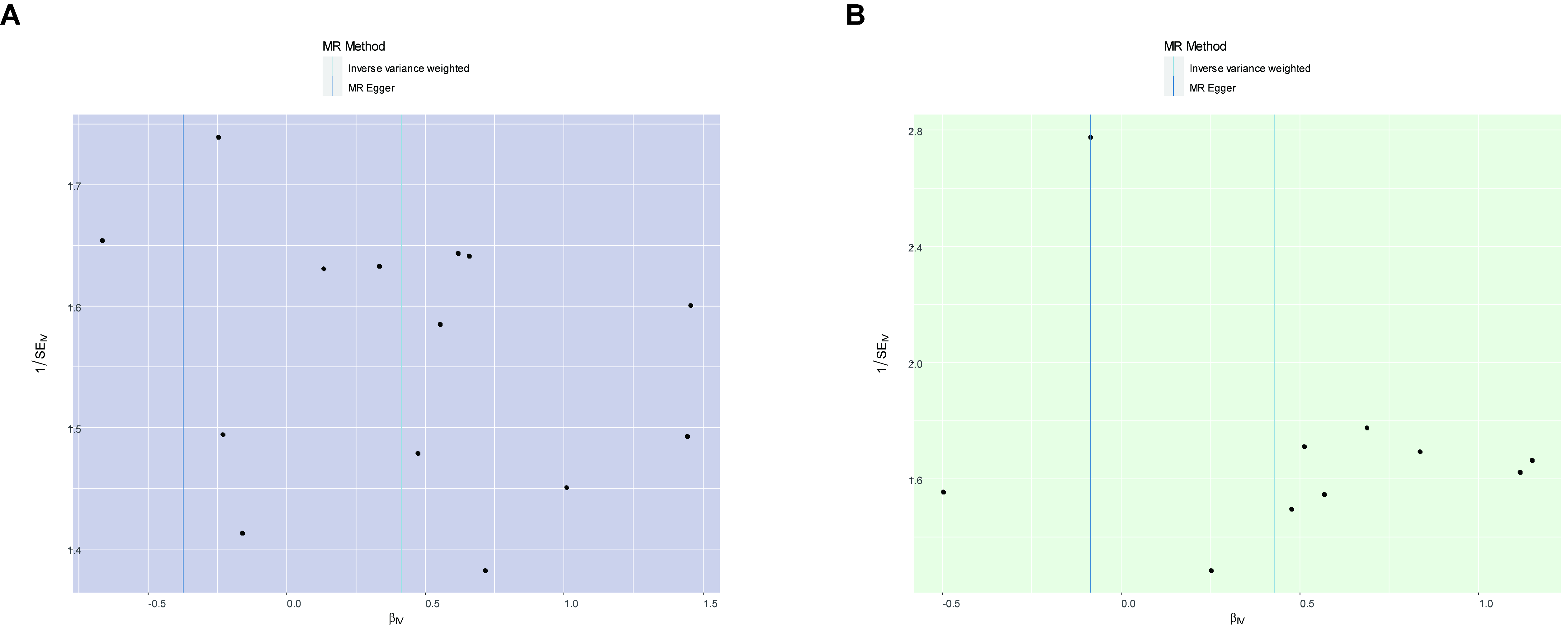

Supplement: S1 Fig — (TIF) [file pone.0299536.s001.tif]
