# Supplementary material for: Causal effect of porphyria biomarkers on alcohol-related hepatocellular carcinoma through Mendelian Randomization
Source: PLoS One. 2024 Mar 20;19(3):e0299536. doi: 10.1371/journal.pone.0299536 (PMC10954128; doi:10.1371/journal.pone.0299536)
Supplement: S1 Table — (DOCX) [file pone.0299536.s002.docx]

Article title: Casual effect of porphyria biomarkers on alcohol-related hepatocellular carcinoma through Mendelian Randomization

Journal name: Journal of cancer research and clinical oncology

Author names: Xiaoyu Yang^12^, Shuomin Wang^12^, Chen Sun^12^ and Yunhong Xia^12*^

^1^Department of Oncology, the First Affiliated Hospital of Anhui Medical University, Hefei, Anhui, China.

^2^Department of Oncology, Anhui Public Health Clinical Center, Hefei, Anhui, China.

*Corresponding author

E-mail: yhxia21@sina.com

**S1 Table. List of GWAS data included in the MR study.**

| Trait | Sample size | N case | N control | Consortium | Year | Population | n SNP |
| --- | --- | --- | --- | --- | --- | --- | --- |
| PBGD | 3,301 | NA | NA | NA | 2018 | European | 10,534,735 |
| UROS | 3,301 | NA | NA | NA | 2018 | European | 10,534,735 |
| AR-HCC | 2,107 | 775 | 1,332 | NA | 2018 | European | 7,800,543 |

GWAS, genome-wide summary association study; MR, Mendelian randomization; PBGD, porphobilinogen deaminase; UROS, uroporphyrinogen-III synthase; AR-HCC, alcohol-related hepatocellular carcinoma; SNP, single-nucleotide polymorphism.
