# Supplementary material for: Causal effect of porphyria biomarkers on alcohol-related hepatocellular carcinoma through Mendelian Randomization
Source: PLoS One. 2024 Mar 20;19(3):e0299536. doi: 10.1371/journal.pone.0299536 (PMC10954128; doi:10.1371/journal.pone.0299536)
Supplement: S2 Table — (DOCX) [file pone.0299536.s003.docx]

Article title: Casual effect of porphyria biomarkers on alcohol-related hepatocellular carcinoma through Mendelian Randomization

Journal name: Journal of cancer research and clinical oncology

Author names: Xiaoyu Yang^12^, Shuomin Wang^12^, Chen Sun^12^ and Yunhong Xia^12*^

^1^Department of Oncology, the First Affiliated Hospital of Anhui Medical University, Hefei, Anhui, China.

^2^Department of Oncology, Anhui Public Health Clinical Center, Hefei, Anhui, China.

*Corresponding author

E-mail: yhxia21@sina.com

**S2 Table. List of SNPs used as IVs in the MR study of biomarkers**

| SNP | Chr | EA | NEA | BETA | SE | id.exposure | F statistic |
| --- | --- | --- | --- | --- | --- | --- | --- |
| rs1927184 | 1 | A | G | 0.118 | 0.026 | prot-a-1348 | 20 |
| rs79548525 | 1 | G | A | -0.289 | 0.065 | prot-a-1348 | 20 |
| rs4149952 | 1 | G | T | 0.361 | 0.078 | prot-a-1348 | 22 |
| rs7532894 | 1 | C | T | -0.185 | 0.042 | prot-a-3176 | 20 |
| rs11925735 | 3 | A | G | 0.122 | 0.027 | prot-a-1348 | 21 |
| rs78217608 | 3 | T | C | 0.316 | 0.071 | prot-a-3176 | 20 |
| rs62315047 | 4 | A | C | 0.299 | 0.065 | prot-a-1348 | 21 |
| rs139639303 | 4 | A | T | 0.452 | 0.099 | prot-a-3176 | 21 |
| rs62313022 | 4 | A | T | 0.222 | 0.049 | prot-a-3176 | 21 |
| rs700715 | 5 | C | T | -0.120 | 0.026 | prot-a-1348 | 21 |
| rs117485699 | 6 | T | A | 0.334 | 0.075 | prot-a-1348 | 20 |
| rs34798299 | 6 | C | G | -0.269 | 0.059 | prot-a-3176 | 21 |
| rs145022107 | 7 | A | C | 0.616 | 0.136 | prot-a-1348 | 21 |
| rs56106813 | 7 | T | C | -0.233 | 0.051 | prot-a-1348 | 21 |
| rs1859686 | 7 | C | T | 0.157 | 0.034 | prot-a-1348 | 21 |
| rs9655993 | 7 | A | C | 0.191 | 0.042 | prot-a-1348 | 21 |
| rs6947681 | 7 | A | G | 0.144 | 0.030 | prot-a-3176 | 23 |
| rs1685815 | 7 | A | G | -0.133 | 0.030 | prot-a-3176 | 20 |
| rs2115807 | 8 | G | C | -0.246 | 0.055 | prot-a-1348 | 20 |
| rs573759343 | 8 | A | T | 0.205 | 0.045 | prot-a-1348 | 21 |
| rs2606196 | 8 | C | T | 0.288 | 0.063 | prot-a-1348 | 21 |
| rs77260147 | 8 | C | G | 0.422 | 0.087 | prot-a-3176 | 24 |
| rs183527459 | 8 | A | G | -0.474 | 0.102 | prot-a-3176 | 22 |
| rs79983112 | 8 | G | A | -0.307 | 0.068 | prot-a-3176 | 20 |
| rs12545798 | 8 | G | T | -0.348 | 0.074 | prot-a-3176 | 22 |
| rs2606196 | 8 | C | T | 0.310 | 0.063 | prot-a-3176 | 25 |
| rs28505556 | 9 | G | A | -0.124 | 0.025 | prot-a-3176 | 25 |
| rs201498139 | 10 | G | A | -0.300 | 0.068 | prot-a-3176 | 20 |
| rs10794029 | 10 | G | A | -0.233 | 0.031 | prot-a-3176 | 56 |
| rs148150385 | 11 | G | C | -0.319 | 0.070 | prot-a-1348 | 21 |
| rs542522683 | 11 | T | G | -0.257 | 0.053 | prot-a-1348 | 24 |
| rs12576116 | 11 | G | C | -0.258 | 0.057 | prot-a-3176 | 20 |
| rs12821733 | 12 | C | A | -0.179 | 0.039 | prot-a-1348 | 21 |
| rs537830342 | 12 | C | T | 0.452 | 0.096 | prot-a-3176 | 22 |
| rs1824843 | 13 | C | T | -0.382 | 0.083 | prot-a-3176 | 21 |
| rs75900623 | 15 | T | C | 0.406 | 0.086 | prot-a-3176 | 22 |
| rs17670276 | 16 | A | C | 0.159 | 0.036 | prot-a-1348 | 20 |
| rs149085574 | 17 | A | G | 0.367 | 0.081 | prot-a-1348 | 21 |
| rs7213686 | 17 | C | T | 0.140 | 0.030 | prot-a-1348 | 22 |
| rs11081299 | 18 | G | T | -0.387 | 0.087 | prot-a-3176 | 20 |
| rs10405386 | 19 | A | G | -0.142 | 0.030 | prot-a-3176 | 22 |
| rs8116001 | 20 | C | A | -0.123 | 0.026 | prot-a-1348 | 22 |
| rs8140987 | 22 | T | C | 0.241 | 0.053 | prot-a-1348 | 21 |
| rs149085574 | 17 | A | G | 0.367 | 0.081 | prot-a-1348 | 21 |
| rs7213686 | 17 | C | T | 0.140 | 0.030 | prot-a-1348 | 22 |
| rs11081299 | 18 | G | T | -0.387 | 0.087 | prot-a-3176 | 20 |
| rs10405386 | 19 | A | G | -0.142 | 0.030 | prot-a-3176 | 22 |

SNP, single-nucleotide polymorphism; IVs, instrumental variables; MR, Mendelian randomization; Chr, chromosome; EA, effect allele; NEA, non-effect allele; SE, standard error.
