# Supplementary material for: Causal effect of porphyria biomarkers on alcohol-related hepatocellular carcinoma through Mendelian Randomization
Source: PLoS One. 2024 Mar 20;19(3):e0299536. doi: 10.1371/journal.pone.0299536 (PMC10954128; doi:10.1371/journal.pone.0299536)
Supplement: Attachment — Submitted filename: Reviewer comments.docx [file pone.0299536.s004.docx]

December 22, 2023

Dear Editor,

I thank you for asking me to review the manuscript titled “Causal effect of porphyria biomarkers on alcohol-related hepatocellular carcinoma through Mendelian Randomization”. The authors have used a novel epidemiological method called Mendelian Randomization to infer causal association of porphyria biomarkers and alcohol-related liver cancer using genome-wide association studies (GWAS) to identify candidate single-nucleotide polymorphisms (SNPs).

1. Novelty: this study fulfils criteria for novelty
2. Biological plausibility: The acute hepatic porphyrias are associated with a risk of primary liver cancer especially among older patients of 50 years with active porphyria. Previous studies (ref 14 & 15) have shown that risk of primary liver cancer is increased in symptomatic patients with Acute Intermittent Porphyria (AIP) due to accumulation of porphyrin precursors.
3. Methodology:
   a. The authors have used a novel technique called Mendelian Randomization: a research method that provides evidence about putative causal relations between modifiable risk factors and disease, using genetic variants as natural experiment. This methodology is less likely to be affected by biases such as confounding or reverse causation.
   b. Although methodology appears sound and supported by literature, it may be worthwhile to have a statistician who is more familiar with Mendelian randomization, MR Egger, inverse variance-weighted and other methods to review it.
4. Conclusion: Conclusion is misleading as nowhere in the manuscript there is description of cases with alcohol-related hepatocellular carcinoma (AR-HCC).

comments:

5. Spelling correction for manuscript title on line 4. It should be “Causal” instead of Casual.
6. Spelling correction on line 26 “porphyria”
7. The authors should remove statement in lines 83-85 of the introduction section, which implies that they have already concluded their hypothesis even before presenting the data.
8. Increased risk of primary liver cancer in AIP and other hepatic porphyrias is well-documented, however it is unclear if congenital erythropoietic porphyria (CEP), an even rare form of cutaneous porphyria, is associated with primary liver cancer. What prompted the authors to look for UROS SNPs in CEP cases?
9. Mechanism of liver injury and progression to AR-HCC in porphyrias was briefly mentioned, it may be worthwhile to expand on it.
10. The authors write that “the causal effect of PBGD and UROS on AR-HCC were confirmed using MR”, as the title also suggests. However, nowhere the causes of HCC are described. If anything, this study establishes a causal associated with HCC but not specifically AR-HCC.
11. Previous studies reported increased urinary PBD in symptomatic patients with AIP and incident cases of AR-HCC. It is unclear from this study whether GWAS cases were symptomatic or had AR-HCC. If that is case, it needs to be stated clearly.
12. Also unclear is why there is increased risk of AR-HCC as opposed to other causes of HCC. This needs to be explained in introduction.

I hope you will find the above comments useful in making decision on this manuscript.

Thank you,

Sajid Jalil, MBBS, MS
